# Antibody-removal therapies for de novo DSA in pediatric intestinal recipients: Why, when, and how? A single-center experience

**DOI:** 10.3389/fped.2022.1074577

**Published:** 2023-02-02

**Authors:** María Lasa-Lázaro, Esther Ramos-Boluda, Esther Mancebo, María José Castro-Panete, Rocío González-Sacristán, Javier Serradilla, Ane Miren Andrés-Moreno, Francisco Hernández-Oliveros, Estela Paz-Artal, Paloma Talayero

**Affiliations:** ^1^ Department of Immunology, University Hospital 12 de Octubre, Instituto de Investigación Sanitaria Hospital 12 de Octubre (imas12), Madrid, Spain; ^2^ Unit of Intestinal Rehabilitation and Transplant, University Hospital La Paz, Madrid, Spain; ^3^ Department of Pediatric Surgery, University Hospital La Paz, Madrid, Spain; ^4^ IdiPaz Research Institute, University Hospital La Paz, Madrid, Spain; ^5^ School of Medicine, Complutense University, Madrid, Spain; ^6^ CIBER de Enfermedades Infecciosas, ISCIII, Madrid, Spain

**Keywords:** intestinal transplantation, multivisceral transplantation, donor-specific antibodies, antibody-removal therapies, pediatric transplantation

## Abstract

**Background:**

Donor-specific anti-HLA antibodies (DSA) impact negatively on the outcome of intestinal grafts. Although the use of antibody-removal therapies (ART) is becoming more frequent in the last few years, issues regarding their timing and effectiveness remain under discussion.

**Methods:**

In the present study, we report our experience with eight ART procedures (based on plasmapheresis, intravenous immunoglobulin, and rituximab) in eight pediatric intestinal and multivisceral transplants with de novo DSA (dnDSA).

**Results:**

ART were performed when dnDSA appeared in two contexts: (1) concomitant with rejection (acute or chronic) or (2) without rejection or any other clinical symptom. Complete DSA removal was observed in seven out of eight patients, showing an effectiveness of 88%. In the group treated for dnDSA without clinical symptoms, the success rate was 100%, with complete DSA removal and without rejection afterward. A shorter time between DSA detection and ART performance appeared as a significant factor for the success of the therapy (*p* = 0.0002). DSA against HLA-A and DQ alleles were the most resistant to ART, whereas anti-DR DSA were the most sensitive. In addition, the 8-year allograft survival rate in recipients undergoing ART was similar to that in those without DSA, being significantly lower in non-treated DSA-positive recipients (*p* = 0.013).

**Conclusion:**

The results confirm the effectiveness of ART in terms of DSA removal and allograft survival and encourage its early use even in the absence of clinical symptoms.

## Introduction

The long-term functionality of intestinal grafts continues to pose a challenge, with the 5-year graft survival rate remaining stagnant at approximately 50% (1). While sepsis and acute cellular rejection are the leading causes of early graft loss and mortality, infections, renal failure, antibody-mediated rejection, and chronic rejection impair long-term graft and patient survival (2).

The deleterious role of anti-HLA donor-specific antibodies (DSA) in intestinal transplantation (ITx) has become more evident in recent years. The presence of de novo DSA (dnDSA) has been associated with decreased allograft survival and a higher incidence and severity of rejection (3–6). Indeed, they have been previously shown as an independent risk factor for 5-year allograft loss with a hazard ratio (HR) of 6.54 (6).

Antibody-removal therapies (ART) in solid organ transplantation include different approaches. Plasmapheresis and intravenous immunoglobulin (IVIG) are usually the first choices of treatment as they quickly remove circulating DSA. The anti-CD20 monoclonal antibody rituximab, which depletes B lymphocytes, is commonly used in combination with the two aforementioned or as second-line therapy. Other strategies for refractory cases include drugs targeting plasma cells (like proteasome inhibitors such as bortezomib and more recently anti-CD38 antibodies), complement molecules (such as the anti-C5 monoclonal antibody eculizumab), or different components of costimulatory pathways (IL6/IL6-receptor antibodies, B7-CD28 complex, etc.) (7–9).

The use of ART in ITx has extended, as evidence of the harmful effects of DSA has increased. Even though ART are used in most centers for preformed DSA (10, 11), there is still no consensus regarding DSA removal in post-transplantation. Although most centers agree on the treatment of the humoral component in the context of acute rejection (both antibody-mediated rejection alone and mixed cellular/humoral rejection), ART in the absence of clinical symptoms remain controversial. The reasons to delay treatment are several, from technical problems stemming from the need for central vein access to logistic issues such as hospitalization or the possibility of infectious complications due to B cell depletion.

In this study, we perform a retrospective analysis of the ART experience of our pediatric ITx cohort for dnDSA, with a focus on the factors that may influence the outcome, and also the effectiveness in terms of graft survival.

## Materials and methods

### Study cohort

**Figure 1:**
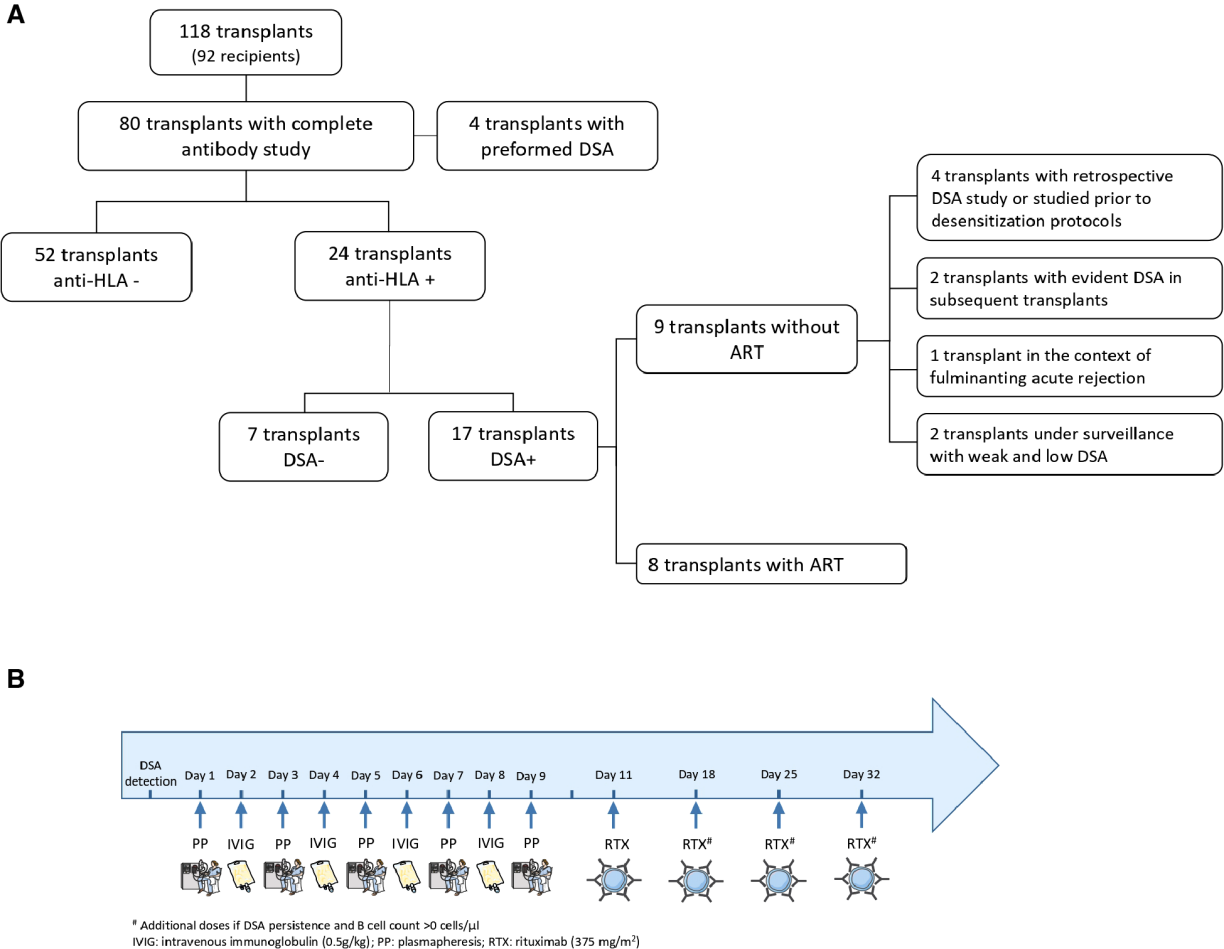
(**A**) Description of the studied cohort. (**B**) Antibody-removal protocol for de novo DSA.

The study included a cohort of 92 recipients of 118 transplants performed at the University Hospital La Paz between 1999 and 2021. This cohort was partially described previously by our group (6). Transplants without a DSA study or those in which donor HLA typing was unknown were excluded from the analysis. Transplants with preformed DSA were also excluded in order to study “pure” dnDSA recipients. Eventually, a total of 76 transplants from 67 patients were included (Figure 1A).

The median age at transplant of this final cohort was 3.3 years (Table 1), with a median follow-up time of 6.9 years. The primary causes of transplantation were short bowel syndrome (48 transplants), motility disorders (13 transplants), congenital mucosal disorders (11 transplants), and others (4 transplants). A total of 59 transplants were first transplants, while 17 were retransplants. A total of 63 transplants included the liver (11 liver intestine and 52 multivisceral), whereas 13 were liver-excluded grafts (10 isolated intestinal grafts and 3 modified multivisceral).

**Table 1:** Demographic and clinical characteristics of the final studied cohort.

	Total (*n* = 76)	DSAneg (*n* = 59)	noART-DSA (*n* = 9)	ART-DSA (*n* = 8)	ART-DSA vs. DSAneg	ART-DSA vs. noART-DSA
Recipient age at transplant, median of years (min–max)	3.3 (0.5–30.1)	3.0 (0.5–30.1)	3.6 (1.8–18)	8.5 (0.7–19.6)	0.05	0.19
Recipient sex, female	29 (38%)	22 (37%)	3 (33%)	4 (50%)	1	0.64
Underlying disease					0.11	0.18
Short bowel syndrome	48 (64%)	40 (68%)	3 (33%)	5 (63%)		
Motility disorders	13 (17%)	7 (12%)	3 (33%)	3 (37%)		
Congenital mucosal disorders	11 (14%)	8 (13%)	3 (33%)	0		
Other	4 (5%)	4 (7%)	0	0		
Previous transplants	17 (22%)	12 (20%)	2 (22%)	3 (37%)	0.36	0.62
Liver-inclusive allograft	63 (83%)	54 (92%)	3 (33%)	6 (75%)	0.19	0.15
Induction therapy					0.43	0.34
Basiliximab	52 (69%)	42 (71%)	5 (56%)	5 (63%)		
Alemtuzumab	17 (22%)	12 (20%)	2 (22%)	3 (37%)		
Thymoglobulin	7 (9%)	5 (9%)	2 (22%)	0		
Maintenance therapy					1	1
Tacrolimus	36 (47%)	27 (46%)	5 (56%)	4 (50%)		
Sirolimus (±tacrolimus)	40 (53%)	32 (54%)	4 (44%)	4 (50%)		

ART, antibody-removal therapy; ART-DSA, DSA-positive recipients undergoing ART; DSAneg, DSA-negative recipients; noART-DSA, DSA-positive recipients not undergoing ART.

### Immunosuppression and ART protocol

Different induction protocols were used as previously reported (12) (Table 1). Maintenance immunosuppression was based on tacrolimus (blood levels of 10–15 ng/ml until the 3rd month and 5–10 ng/ml from the 3rd month onward). When induction was made with basiliximab, corticosteroids were also required as part of maintenance therapy. Sirolimus (2 mg/m^2^ daily with blood levels in the 5–10 ng/ml range) was used according to what was previously reported by our group (12) in 40 transplants (8 combined with tacrolimus and 32 in monotherapy).

The standard post-transplant ART protocol consisted of five plasmapheresis sessions together with four doses of 0.5 g/kg IVIG (a total of 2 g/kg) on alternate days. Rituximab was administered 24–48 h later in boluses of 375 mg/m^2^ once a week up to a maximum of 4 weeks (Figure 1B). ART was initiated when DSA with a mean fluorescence intensity (MFI) higher than 2000 appeared.

Graft monitoring was based on symptoms, and endoscopic biopsies were performed only when rejection was clinically suspected. Rejection diagnosis was made on the basis of both clinical and histological data. Clinical symptoms were fever, nausea, vomiting, diarrhea, abdominal pain and distension, and increased stomal effluent volume. A histological diagnosis (with at least two independent pathologists) was made according to previously reported criteria (13, 14).

### Anti-HLA antibody study

Sera for the period before 2010 were collected pre-transplant and studied retrospectively. From 2010 onward, they were prospectively analyzed before transplant, at 15 days post-transplant, 1 month, 3 months, 6 months, and every 6 months thereafter, and when clinical events appeared.

Anti-HLA antibodies were tested by using Luminex Technology. A LABScreen Mix (LSM) kit (One Lambda, CA, USA) was used for the initial screening. The test was considered positive when standard fluorescence intensity (SFI) was more than 15.000 for anti-HLA class I or above 20.000 for anti-HLA class II. Specificities were determined by using a LABScreen Single Antigen (LSA) kit (One Lambda, CA, USA). An MFI of over 500 was considered positive. The C1q binding assay was performed with a C1qScreen kit (One Lambda, CA, USA). The test was considered positive when the MFI was more than 500.

### Statistical analysis

A statistical analysis of categorical data frequencies was carried out using Fisher's exact test or a Chi-square test. The significance of differences when comparing quantitative data was determined using the Mann–Whitney *U* test. For correlation analysis, Spearman's Rho was calculated. Kaplan–Meier and log-rank tests were used to perform survival analysis among groups. *P*-values <0.05 were considered significantly different. GraphPad Prism software (version 8.0.2) was used for the analysis.

## Results

### ART indications and protocols

From the final cohort of 76 transplants, dnDSA were detected in 17 patients. Of these, nine did not undergo ART (noART-DSA) because of different reasons (Figure 1A) and eight DSA-positive transplants, underwent ART (Table 2). Six recipients had multivisceral grafts and two had isolated intestinal grafts. No significant differences were observed in the recipients' clinical characteristics between this group and the DSAneg and noART-DSA groups (Table 1).

**Table 2:** Summary of the ART carried out.

ID	N. Tx	Graft type	Indication	Protocol	HLA Class	LSM^a^	LSA^b^	C1q	DSA removal
P1	2	ISBT	De novo with chronic rejection	IVIG + PP + RTX	Anti HLA-I	+	+++	*P*	No
					Anti HLA-II	+	+++	*P*	No
P2	1	ISBT	De novo with chronic rejection	IVIG + RTX	Anti HLA-II	++	+++	*P*	Yes
P3	1	MVT	De novo with severe acute rejection	IVIG + CE + TMG	Anti HLA-I	+	+++	*P*	Yes
					Anti HLA-II	+	+++	*P*	Yes
P4	2	MVT	De novo without rejection	IVIG + PP + RTX	Anti HLA-II	+++	+++	N	Yes
P5	1	MVT	De novo without rejection	IVIG + PP + RTX	Anti HLA-I	+	+	N	Yes
					Anti HLA-II	+	+++	N	Yes
P6	2	MVT	De novo without rejection	IVIG + RTX	Anti HLA-II	++	++	N	Yes
P7	1	MVT	De novo without rejection	IVIG + PP + RTX	Anti HLA-I	+	+++	*P*	Yes
					Anti HLA-II	+	+++	*P*	Yes
P8	1	MVT	De novo without rejection	IVIG + PP + RTX	Anti HLA-II	+	+++	*P*	Yes

CE, corticosteroids; ISBT, isolated small bowel transplant; IVIG, intravenous immunoglobulin; LSA, LABScreen Single Antigen; LSM, LABScreen Mix; MVT, multivisceral transplant; N, negative; N. Tx, number of transplant; *P*, positive; PP, plasmapheresis; PTLD, post-transplant lymphoproliferative disorders; RTX, Rituximab; TMG, thymoglobulin.

^a^ +++: > 350,000 SFI; ++: 150,000–350,000 SFI; +: 15,000–150,000 SFI.

^b^ +++: > 4,000 MFI; ++: 2000–4,000 MFI; +: 500–2,000 MFI. MFI of immunodominant DSA.

ART was administered when dnDSA appeared in either of two scenarios: (1) acute or chronic rejection (*n* = 3) or (2) no rejection or any other clinical symptom (*n* = 5). Altogether, eight ART procedures (matching each one to one transplant from one single recipient) were performed (Table 2).

In five procedures, the standard therapy with plasmapheresis, IVIG, and rituximab (Table 2, Figure 1B) was used. In another two procedures, IVIG and rituximab were used without plasmapheresis because of the difficulty in gaining access to the central vein. Only in the recipient with dnDSA concomitant to an acute rejection episode (P3), the therapy was changed (IVIG plus thymoglobulin and corticosteroids) in order to also treat the rejection cellular component.

### ART was effective in 88% of the recipients

We first defined ART effectiveness in terms of DSA removal as follows: (A) a full negativization of anti-HLA antibodies (LSM negative) or (B) no DSA detection in LSA (DSA MFI <500), maintained at least for 1 year in both cases.

**Figure 2:**
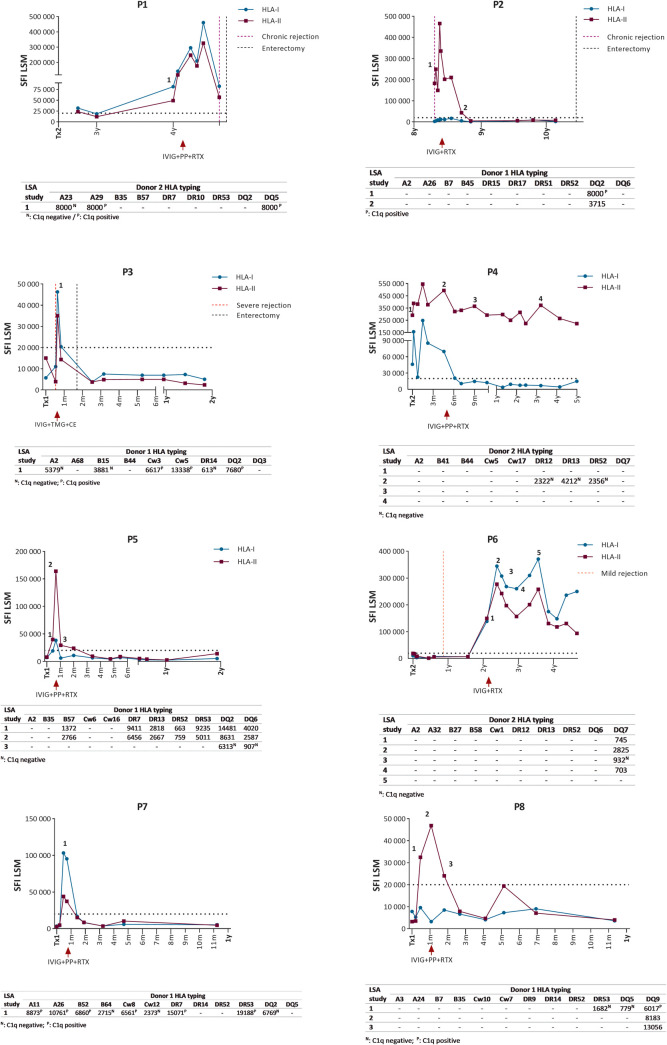
Evolution of anti-HLA antibodies levels (SFI) and DSA MFI in the eight treated recipients. Antibody-removal procedures and important clinical events are represented. CE, corticosteroids; HLA-I, anti-HLA class-I antibodies; HLA-II, anti-HLA class-II antibodies; IVIG, intravenous immunoglobulin; LSA, LABScreen single antigen; LSM, LABScreen Mix; PP, plasmapheresis; RTX, rituximab; TMG, thymoglobulin.

In the context of dnDSA appearance concomitant with rejection, ART was administered to three recipients. In the first patient (P1), DSA monitoring started 3 years after transplantation, revealing then the existence of DSA vs. A23, A29, and DQ5 with positivity for C1q assay for A29 and DQ5 (Figure 2). However, she was not treated until chronic rejection was diagnosed 1 year later. Then, the standard therapy with plasmapheresis, IVIG, and rituximab was applied, however, with no effect, as there was no increase in antibody levels or no clinical improvement, with only subsequent graft loss.

Patient 2 was first monitored for DSA in the context of a chronic 8-year post-transplantation, and complement-fixing DSA vs. DQ2 was noted. For this reason, she was treated with IVIG and rituximab (no plasmapheresis was done because of a lack of vein access), and although DSA quickly disappeared, allograft loss could not be avoided.

Patient 3 suffered from a severe acute rejection in the early post-transplant period with high MFI DSA vs. multiple specificities, several of them being C1q-positive (Figure 2). He was treated with corticosteroid, thymoglobulin, and IVIG, achieving a fast DSA clearance. Nonetheless, intestinal graft loss was unavoidable, and he was enterectomized, however keeping the liver, pancreas, stomach, and duodenum grafts intact.

Therefore, although DSA removal was done in two out of the three recipients from this group (67% effectiveness), graft loss could not be avoided in any of the cases.

In five more patients (P4, P5, P6, P7, and P8), ART was administered because of dnDSA appearance in the absence of any clinical events or rejection signs. All DSA were removed in all patients (100% effectiveness) and no rejection occurred in any of the recipients after ART. No rebound was observed in any patient, and the median follow-up time was 2 years (1–6.2 years).

Therefore, considering all recipients together, ART was effective in 88% of them (7/8) (Table 2).

### Time from DSA detection to ART is a significant factor for a successful outcome

**Figure 3:**
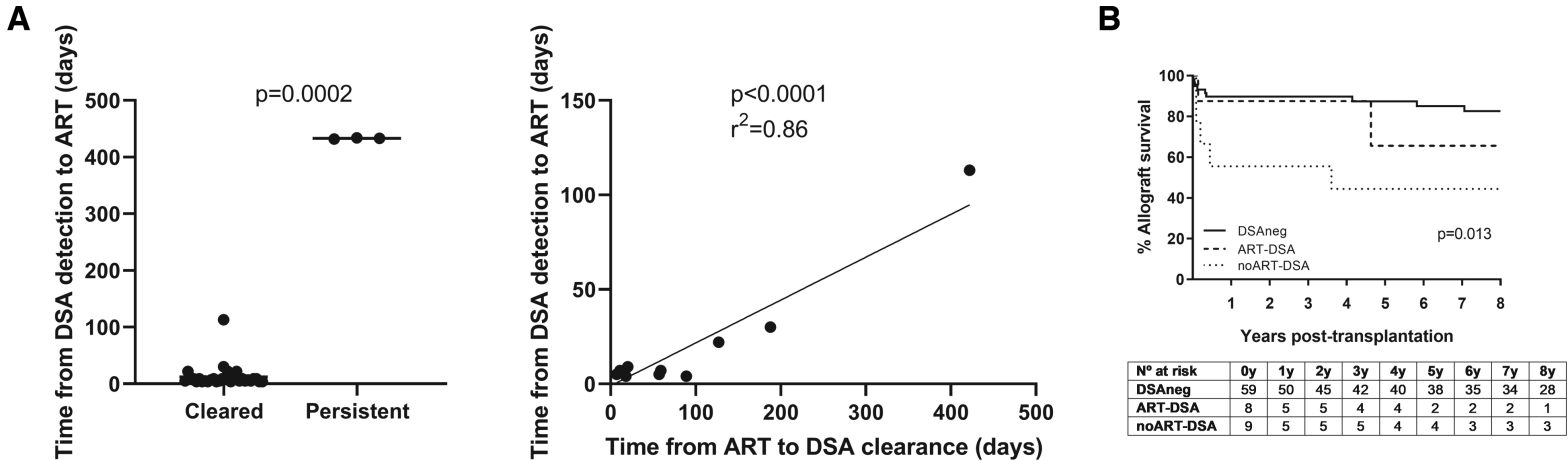
(**A**) Analysis of the elapsed time between DSA detection and antibody-removal procedure: comparison in cleared and persistent DSA and correlation with the time until DSA clearance. (**B**) Eight-year graft survival analysis in DSAneg, ART-DSA, and noART-DSA recipients.

An important issue under debate is the timing of ART and especially when DSA are present with good graft function. Because we observed that in patients with delayed ART, DSA were more resistant, we analyzed whether the time from DSA detection to therapy performance had any impact on the outcome. We observed that the time was significantly shorter for cleared than for persistent DSA (7 days vs. 433 days; *p* = 0.0002; Figure 3A), and it also significantly correlated with the time until DSA clearance (*p *= <0.0001; *r*^2 ^= 0.86; Figure 3A).

Analyzing other possible factors (previously reported by others) such as HLA specificity or DSA intensity, we found that DSA against HLA-A alleles were the most resistant (60% of clearance), while DQ alleles were the ones that took the longest time to get cleared (with a 89% clearance rate and a median clearance time of 74 days). Anti-DR DSA were the most sensitive to ART (with a 100% clearance rate and a median time of 18 days) (Supplementary Table S1). As far as MFI was concerned, it was higher for persistent than for cleared DSA (median MFI: persistent = 8,354, cleared = 5,698), although this difference was not significant due to the low number of persistent DSA (*p* = 0.21).

### Long-term allograft survival rate in treated recipients is similar to that in DSA-negative patients

After analyzing ART effectiveness in terms of DSA removal, we wanted to determine whether it had any impact on graft survival. The starkest contrast was observed at 1-year post-transplantation, with a 90% survival rate in the DSAneg and ART-DSA groups but only 50% in noART-DSA recipients (Figure 3B). Significant differences among the three groups were also maintained in the long term (*p* = 0.013), with 8-year allograft survival rate in DSAneg recipients being similar to that in ART-DSA patients (*p* = 0.37) but not to that in the noART-DSA group (*p* = 0.0033). Importantly, DSA characteristics were similar between the ART-DSA and the noART-DSA groups (Supplementary Table S2).

It is well known that one of the main factors affecting graft survival is rejection. In our ART-DSA group, the rejection rate before the therapy was 37.5% (Table 3), which belonged to the three transplants in which dnDSA appeared together with rejection (P1, P2, and P3). After excluding these three patients who lost their grafts and then the rejection rate after the ART could not be evaluated, no rejection episodes were observed in the remaining five cases. This rejection rate strongly differed from that observed in the noART-DSA group (*p* = 0.021).

**Table 3:** Rejection rates in different recipient groups.

** **	Rejection rate	*p*-Value vs. DSAneg
DSAneg	24% (14/59)	
noART-DSA	78% (7/9)	0.0028
ART-DSA pre-ART	38% (3/8)	0.41
ART-DSA post-ART	0% (0/5)	0.58

With regard to possible complications after ART, only one patient suffered from a catheter-associated bacteremia 1 month after the therapy. No further complications were observed, which underlined the safety of ART.

## Discussion

The harmful effect of DSA (either preformed, de novo, or persistent) on ITx has been described in the last few years by several groups (3–5). Our previous report including 43 transplants from 36 recipients showed a deleterious impact of dnDSA on graft rejection (HR = 10.39) and survival (HR = 66.52), being an independent risk factor for 5-year allograft loss (6).

As this evidence emerged, ART was implemented. Most centers agree on the use of plasmapheresis, IVIG, and rituximab for humoral component treatment in the context of rejection (15–17). Nevertheless, ART in the absence of clinical symptoms remains controversial. While some groups support the idea of dnDSA removal soon after their appearance (15, 16) or similar treatment for strong DSA (5, 18), some centers are still reluctant to carry out such procedures without the appearance of any signs of rejection. The limited experience with the lack and disparity of information about ART indications and outcomes and technical, logistic, and clinical complications arising from the treatment are the main reasons for delaying such therapies.

In our cohort, preformed antibodies in the first transplant was mostly absent, with only 1 patient case reported from the 64 studied (data were unavailable for the remaining cases), which meant that, except in this one patient, all DSA were generated after transplantation. When we began DSA monitoring, we actually observed that DSA were mostly present in those patients with previous transplants and who had experienced severe and/or chronic rejection; however, we were not able to ascertain whether there were any causes or whether they denoted consequences. With routine DSA determination, we started to identify their correlation with chronic rejection either before their appearance (P1) or when they were already established (P2). Because these two recipients belonged to the initial period of DSA monitoring and ART, DSA were detected late (with no exact knowledge about their appearance) and chronic rejection and allograft loss could not be avoided. In another patient (P3), dnDSA appearance was observed coincident to a severe rejection episode, and this case was a clear consequence of T-cell activation. In contrast to what was reported by Gerlach (15) and Wozniak (19), who identified that dnDSA mostly appeared in the context of acute cellular rejection episodes, we observed a higher proportion of patients in whom dnDSA emerged without any clinical events, which is currently the most frequent trend in our cohort among new cases. In such patients and due to our previous experience, which related persistent DSA to chronic allograft loss, ART was performed for dnDSA with >2,000 MFI with excellent results and complete removal in 100% of patients.

In this study, we aimed to deepen our experience with ART in ITx, attempting to provide additional evidence to answer the “why, when, and how” question.

First addressing the “why”, we found two main scenarios in which ART was performed: (1) in acute rejection episodes with humoral component and (2) when dnDSA appeared without any clinical symptom. Interestingly, therapy success was closely related to indication. Thus, within the first group (dnDSA with rejection), the therapy was effective in two out of three transplants, but allograft loss could not be avoided in any patient. The best results were observed in the second group (dnDSA without rejection), in which ART was successful in all recipients with complete DSA removal, with only one procedure needed and no rejection thereafter.

This fact led us to answer the “when” question. Because we observed that in patients with delayed ART, DSA were more resistant, we analyzed whether the time from DSA detection until ART administration could have any impact on the outcome, and indeed we found a significant correlation. For example, in the patient in whom DSAs were not removed, the time from DSA to ART was the longest. This important factor, which has not been reported before, strongly makes a case for treating DSA soon after detection or as early as possible even in the absence of clinical symptoms.

With regard to the “how” question, we followed a combination based on plasmapheresis, IVIG, and rituximab. However, there is a lack of detailed information about doses in ITx, periodicity, or the drug combinations used. Also, in other solid organs, protocols differ on such matters, with a large number of administrative guidelines (20–23). Our protocol is based on the daily alternate administration of plasmapheresis and high-dose IVIG (24), which may differ from that of others, who first perform a 1-week plasmapheresis cycle, followed by IVIG administration either in low or in high doses. The timing of rituximab administration is another controversial issue, with administration ranging from 24 to 48 h (as we did) to 15 days after the end of IVIG or plasmapheresis (21, 25–27).

The final question to address is, “is it worth it?” In our experience, which showed a patient success rate of 88%, the answer is an outright yes. But, apart from the effectiveness in terms of DSA removal, another relevant finding of this work is the survival analysis showing a long-term allograft survival of ART-DSA transplants similar to those of DSAneg and higher than noART-DSA transplants. These results are in accordance with what was described by Cheng et al. (4), who reported a risk of allograft failure of 10% by 1 year and 28% by 2 years after dnDSA detection. Also, Matsumoto and Rosen-Bronson reported some preliminary data showing the same 1-year allograft survival between desensitized recipients who developed dnDSA vs. those who never developed dnDSA (16). All these facts, together with the low rate of complications observed in our cohort, encourage the use of antibody-removal protocols.

This study has some limitations that should be mentioned. The first is the reduced number of treated recipients since only 47% of DSA patients underwent ART. In light of this, a separate survival analysis according to ART indication (which would have been more accurate) could not be performed because of the small sample size. However, we used the available information as best we could, providing details of each single evolution after the therapy. Another limitation is that noART-DSA transplants mostly belong to a previous era (before ART came into vogue) and are not contemporary to ART-DSA transplants; therefore the possibility of an element of bias in the comparisons made between both groups cannot be ruled out. Finally, second-line drugs were not used because of the effectiveness of ART in our cohort. Therefore, our work does not present a full desensitization protocol that includes second- and third-line therapies.

In conclusion, the present study gathers our single-center experience with ART in pediatric ITx, showing high effectiveness in terms of DSA removal (especially for dnDSA without rejection) and an increase in allograft survival rates. The elapsed time between DSA detection and the start of treatment was a significant factor contributing to therapy success, suggesting that the early administration of ART, even in the absence of clinical symptoms, might be beneficial to patients.

## Data Availability

The raw data supporting the conclusions of this article will be made available by the authors without undue reservation.
