# Supplementary material for: Antibody-removal therapies for de novo DSA in pediatric intestinal recipients: Why, when, and how? A single-center experience
Source: Front Pediatr. 2023 Feb 2;10:1074577. doi: 10.3389/fped.2022.1074577 (PMC9932897; doi:10.3389/fped.2022.1074577)
Supplement: Supplementary file 1 [file Table1.docx]

**Supplementary table 1. DSA clearance according to HLA loci**

| **HLA locus** | **Recipients with DSA** | **N of DSA** | **% C1q+^*^** | **% Clearance** | **Median clearance time (days)** |
| --- | --- | --- | --- | --- | --- |
| **A** | 75% (3/4) | 5 | 60% (3/5) | 60% (3/5) | 20 |
| **B** | 75% (3/4) | 4 | 33% (1/3) | 100% (4/4) | 20 |
| **C** | 50% (2/4) | 4 | 75% (3/4) | 100% (4/4) | 38,5 |
| **DR** | 63% (5/8) | 11 | 29% (2/7) | 100% (11/11) | 18 |
| **DQ** | 88% (7/8) | 9 | 44% (4/9) | 89% (8/9) | 74 |

^*^C1q study was not possible for all DSAs, because some C1q studies were performed in sera negative for those DSAs.

**Supplementary table 2**. DSA characteristics in DSA positive recipients.

|  | **ART-DSA* (n=8)** | **noART-DSA (n=9)** | **p-value** |
| --- | --- | --- | --- |
| **LSM Class I levels**  median SFI (min-max) | 58075 (9570-344369) | 33408 (10000-380143) | 1 |
| **LSM Class II levels**  median SFI (min-max) | 44500 (32473-493732) | 49044 (20459-304056) | 0,37 |
| **Type of DSA** |  |  | 0,35 |
| Anti-HLA class I | 0 | 2 (22%) |  |
| Anti-HLA class II | 4 (50%) | 3 (33%) |  |
| Anti-HLA class I + II | 4 (50%) | 4 (45%) |  |
| **Maximum MFI**  median (min-max) | 8000 (2825-19188) | 3065 (879-18299) | 0,27 |
| * values before every desensitization procedure as listed in Table 2  ART: antibody-removal therapy; ART-DSA: DSA positive recipients undergoing ART; noART-DSA: DSA positive recipients not undergoing ART. | | | |
